# Glimpse of the Epidemiological Research on *Helicobacter Pylori* in Saudi Arabia

**DOI:** 10.4103/1319-3767.48963

**Published:** 2009-04

**Authors:** Abdulaziz A. BinSaeed

**Affiliations:** Department of Family and Community Medicine, KSU Research Chair of Epidemiology and Public Health, College of Medicine, King Saud University, Riyadh, Saudi Arabia

***Helicobacter pylori*** (***H. pylori***) is one of the most common infections that affects humans. Although ***H. pylori*** was only identified in 1983,[1] numerous studies reveal that it is encountered worldwide. It is estimated that half or more of the world's population is infected.[2] Substantial differences in the prevalence of ***H. pylori*** infection have been observed between countries.[2,3] These differences conform to two major patterns that differ in developed and developing countries. The general trend of ***H. pylori*** prevalence in developed countries is slow increases during childhood, which continue through adolescence and early adulthood, with an abrupt increase around 50–60 years of age.[4] In the nonindustrialized countries, ***H. pylori*** prevalence increases more rapidly during childhood and most adolescents and adults are infected. Thus, differences in ***H. pylori*** prevalence between industrialized and nonindustrialized countries are greater at younger ages and get smaller at older ages.[2,3,5] Although the overall prevalence is generally lower in the Western world, high prevalence, approaching that of the nonindustrialized nations, have been observed within some subgroups in the industrialized nations. Differences in prevalence within populations are due to a variety of factors primarily relating to socioeconomic status and geographic origin.

In this issue of this Journal, a report about ***H. pylori*** prevalence and its association with recurrent abdominal pain is published.[6] The authors of this publication have surveyed a group of school children for ***H. pylori*** using the ^14^C urea breath test. They further tried to establish a relationship between ***H. pylori*** and recurrent abdominal pain. In spite of the importance of this report, it has potential methodological problems. Using a radioactive material as a tool of diagnosis in the young agegroup is questionable, especially with the lack of credible evidence that supports that decision. Even in the presence of some references that probably mention its safety to be used in older children,[7] conducting a research on healthy people requires the use of the safest procedure ever, especially with the presence of better alternatives that can be used.[8,9] Other limitations of the report are the lack of very important information on demographic and socioeconomic factors that may explain some of the conflicting findings. These problems are not limited to this paper. Research from the region in this field is limited and most of the published papers that emanated from this region have had inherent problems related to their design and sample size. The issue requires further in-depth investigation in order to address various regional questions of epidemiological importance.

A PubMed^®^ search, using ***H. pylori*** as a keyword, shows a great interest in research of this infection. This interest is reflected by the exponential increase in the number of papers published on the subject from a total of 14 in 1984 to over 500 in 1990 and over 26,000 in 2007, making it one of the most active areas of research in the medical field. The Saudi contribution to this enormous literature is very minimal, with less than 70 publications. In contrast, publications from Australia, with a similar population but a lower prevalence of ***H. pylori***, exceeded 500 articles. In conclusion, the strong effort and commitment of several medical disciplines with a large and proper collection of scientific data from Saudi population/subgroups will give significant support to this field of research.
